# Analysis of clinical characteristics of children with Aicardi-Goutieres syndrome in China

**DOI:** 10.1007/s12519-022-00545-1

**Published:** 2022-05-12

**Authors:** Wei Wang, Wei Wang, Ting-Yan He, Li-Ping Zou, Wen-Dao Li, Zhong-Xun Yu, Ming-Sheng Ma, Jun Yang, Hong-Mei Song

**Affiliations:** 1 Peking Union Medical College Hospital, Chinese Academy of Medical Sciences, Peking Union Medical College, No. 1 Shuaifuyuan Wangfujing, Dongcheng District, Beijing, 100730 China; 2 Shenzhen Children’s Hospital, No. 7019 Yitian Road, Futian District, Shenzhen, 518048 China; 3 The General Hospital of the People’s Liberation Army, No. 28 Fuxing Road, Haidian District, Beijing, 10000 China

**Keywords:** Aicardi-Goutieres syndrome, Chinese, Diagnosis, Manifestations

## Abstract

**Background:**

Aicardi-Goutieres syndrome (AGS) is an inflammatory disorder belonging to the type I interferonopathy group. The clinical diagnosis of AGS is difficult, which can lead to a high mortality rate. Overall, there is a lack of large-sample research data on AGS in China. We aim to summarize the clinical characteristics of Chinese patients with AGS and provide clues for clinical diagnostic.

**Methods:**

The genetic and clinical features of Chinese patients with AGS were collected. Real-time polymerase chain reaction was used to detect expression of interferon-stimulated genes (*ISGs*)*.*

**Results:**

A total of 23 cases were included, consisting of 7 cases of AGS1 with three prime repair exonuclease 1 mutations, 3 of AGS2 with ribonuclease H2 subunit B (*RNASEH2B*) mutations, 3 of ASG3 with *RNASEH2C*, 1 of AGS4 with *RNASEH2A* mutations, 2 of AGS6 with adenosine deaminase acting on RNA 1 mutations, and 7 of AGS7 with interferon induced with helicase C domain 1 mutations. Onset before the age of 3 years occurred in 82.6%. Neurologic involvement was most common (100%), including signs of intracranial calcification which mainly distributed in the bilateral basal ganglia, leukodystrophy, dystonia, epilepsy, brain atrophy and dysphagia. Intellectual disability, language disability and motor skill impairment were also observed. Skin manifestations (60.87%) were dominated by a chilblain-like rash. Features such as microcephaly (47.62%), short stature (52.38%), liver dysfunction (42.11%), thyroid dysfunction (46.15%), positive autoimmune antibodies (66.67%), and elevated erythrocyte sedimentation rate (53.85%) were also found. The phenotypes of 2 cases fulfilled the diagnostic criteria for systemic lupus erythaematosus (SLE). One death was recorded. *ISGs* expression were elevated.

**Conclusions:**

AGS is a systemic disease that causes sequelae and mortality. A diagnosis of AGS should be considered for patients who have an early onset of chilblain-like rash, intracranial calcification, leukodystrophy, dystonia, developmental delay, positive autoimmune antibodies, and elevated *ISGs*, and for those diagnosed with SLE with atypical presentation who are nonresponsive to conventional treatments. Comprehensive assessment of vital organ function and symptomatic treatment are important.

## Introduction

Aicardi-Goutieres syndrome (AGS) (OMIM 225750), first reported in 1984 [1], is a genetically determined early-onset encephalopathy with a variable phenotype. Typical clinical manifestations are progressively worsening neurological symptoms, spasticity and dystonia, microcephaly, and severe developmental delay, among others, which are similar to congenital viral infections in the neonatal period. Other clinical manifestations include thrombocytopaenia, hepatosplenomegaly and mild elevation of liver enzymes. The mortality rate of AGS is approximately 19.3% [2]. Seven genes have been reported to result in the classical AGS phenotype: three prime repair exonuclease 1 (*TREX1*; AGS1), ribonuclease H2 subunit B (*RNASEH2B*; AGS2), *RNASEH2C* (AGS3), *RNASEH2A* (AGS4), SAM and HD domain containing triphosphate triphosphohydrolase 1 (*SAMHD1*; AGS5), adenosine deaminase acting on RNA (*ADAR1*; AGS6) and interferon induced with helicase C domain 1 (*IFIH1*; AGS7) [3–6]. In addition, genes such as *LSM11* [7], U7 small nuclear 1 [7], and polyribonucleotide nucleotidyltransferase 1 [8] were reported to be associated with AGS. AGS 2–5 are inherited in an autosomal recessive manner, AGS1 and 6 in an autosomal recessive or autosomal dominant manner, and AGS7 in autosomal dominant manner. In 2011, AGS was listed as a type I interferonopathy by the International Union of Immunological Societies Expert Committee [9].

The number of AGS patients in China is small, and no clear data are available to date. Furthermore, there is no clear standard for the clinical diagnosis of AGS, and misdiagnosis and untimely treatment occur. Some patients with AGS have been reported to be treated effectively [10, 11]. Janus kinase inhibitors have been used to treated AGS and new therapeutic approaches, such as reverse transcriptase inhibitors have been recently proposed, which may be effective to treat patients with mutations of *TREX1*, *RNASEH2A*, *RNASEH2B* and *SAMHD1* [12]. Early detection is of great significance for early diagnosis and early treatment, reducing mortality and improving prognosis. This study aims to summarize and analyse the clinical characteristics of patients with AGS in China and to provide clues for the clinical identification and diagnosis of AGS.

## Methods

This study was approved by the Ethics Committees of Peking Union Medical College Hospital (JS-1660). Informed consents to participate in the study have been obtained from participants (or their parent or legal guardian in the case of children under 16).

Twenty-three Chinese patients with genetically confirmed AGS were enrolled, among whom 8 were diagnosed by the Department of Pediatrics of Peking Union Medical College Hospital and 5 by the Department of Rheumatology and Immunology of Shenzhen Children's Hospital. The remaining 10 cases were obtained from a literature review. We searched PubMed using the term “Aicardi-Goutieres syndrome AND China” and the Wanfang database using the term “Aicardi-Goutieres syndrome” from January 1984 to March 2021. A total of 41 relevant articles were identified, among which 40 studies remained after duplicates. Next, we excluded articles about basic experiments (17), animal trials (4), reviews (6), unrelated diseases (2) and studies without sufficient data for analysis (1). The remaining ten articles involved 12 patients. As we removed 2 duplicate cases, 10 remained.

According to the literatures, neurologic phenotypes of AGS can be classified as prenatal onset AGS (onset of disease in utero), infantile onset AGS (0–12 months), later onset AGS (> 12 months), bilateral striatal necrosis (related with mutations of *ADAR1* gene), hereditary spastic paraparesis (without any other symptoms), *SAMHD1*-related cerebrovascular disease [13]. The sex, age of onset, clinical characteristics, biological parameters, imaging examination, and genetic analysis results of the 23 patients were recorded. The reference ranges of the related laboratory indexes have been shown in Table 1.

**Table 1:** Reference ranges of the related laboratory indexes

Laboratory indexes	Unit	Reference ranges
Liver enzymes		
ALT	U/L	9–50
AST	U/L	15–40
Complement		
C3 complement	g/L	0.73–1.46
C4 complement	g/L	0.1–0.4
Autoantibodies		
Anti-nuclear antibody		< 1:80 (immunofluorescence)
Anti-double-stranded DNA antibody		< 1:10 (immunofluorescence)
Rheumatoid factor	IU/mL	0–20
Anti-neutrophil cytoplasmic antibody		< 1:10 (immunofluorescence)
Anti-Sjogren syndrome A antibody		< 15 (western blot) < 1:1 (double diffusion)
Anti-Sjogren syndrome B antibody		< 15 (western blot) < 1:1 (double diffusion)

*ALT* alanine aminotransferase, *AST* aspartate aminotransferase

Real-time polymerase chain reaction (PCR) was used to detect the expression of interferon-stimulated genes (*ISGs*), including interferon alpha-inducible protein 27 encoding gene (*IFI27*), *IFI44L*, *IFIT1*, *ISG15*, and radical S-adenosyl methionine domain containing 2 encoding gene (*RSAD2*). The primers were designed by the Primer 3.0 web-based server to amplify *ISGs* as Table 2. Using TRIzol reagent, total RNA was extracted from whole blood. Taking 200 ng of total RNA as the template, quantitative reverse transcription PCR analysis was performed using a PrimerScript™ RT Reagent Kit with gDNA Eraser (Takara, Japan) according to the manufacturer's protocols. Then, real-time quantitative PCR was performed using TB Green™ Premix Ex Taq™ II Tli RNaseH Plus following the manufacturer's recommendations on an Applied Biosystems 7500 Fast Real-Time PCR System. The thermal profile was set at 1 cycle at 95 °C for 30 seconds, followed by 40 cycles at 95 °C for 3 seconds and 60 °C for 30 seconds. The relative abundance of each target transcript was normalized to the expression level of β-actin. The relative quantification (RQ) calculations were performed using the comparative CT method (ΔΔCT). The RQ for each transcript is equal to 2^− ΔΔ*C*T^.

**Table 2:** Primers of the interferon-stimulated genes

Genes	Forward (5ʹ–3ʹ)	Reverse (5ʹ–3ʹ)
*IFI27*	TGCTCTCACCTCATCAGCAGT	CACAACTCCTCCAATCACAACT
*IFI44L*	TTGTGTGACACTATGGGGCTA	GAATGCTCAGGTGTAATTGGTTT
*IFIT1*	AGAAGCAGGCAATCACAGAAAA	CTGAAACCGACCATAGTGGAAAT
*ISG15*	GAGGCAGCGAACTCATCTTT	AGCATCTTCACCGTCAGGTC
*RSAD2*	TGCTTTTGCTTAAGGAAGCTG	AGGTATTCTCCCCGGTCTTG
*Actin*	CCAACCGCGAGAAGATGA	CCAGAGGCGTACAGGGATAG

*IFI* interferon alpha-inducible protein encoding gene, *ISG* interferon-stimulated gene, *RSAD* radical S-adenosyl methionine domain containing 2 encoding gene

## Results

### Patient characteristics

A total of 23 patients with AGS from 20 families were enrolled, including 7 (30.43%) cases of AGS1, 3 (13.04%) cases of AGS2, 3 (13.04%) cases of ASG3, 1 (4.35%) case of AGS4, 2 (8.70%) cases of AGS6, and 7 (30.43%) cases of AGS7 (Table 3).

**Table 3:** Gene mutations

Family	Patient	Gene	Mutation	Diagnosis
F1	P1^a^	*TREX1*	**c.505C > T, p.R169C** c.900delA, p.S301Lfs*31	AGS1
F2	P2^a^	*TREX1*	**c.139G > A, p.G47S** c.458dupA, p.C154Mfs*3	AGS1
F3	P3^a^	*TREX1*	c.459dupA, p.C154Mfs*3 c.695delA, p.Y232Sfs*3	AGS1
F4	P4 [32] Sister of P5	*TREX1*	c.227C > T, p.A76V c.458dupA, p.Q153fs*3	AGS1
F4	P5 [32] Brother of P4	*TREX1*	c.227C > T, p.A76V c.458dupA, p.Q153fs*3	AGS1
F5	P6 [14]	*TREX1*	**c.45G > T, p.R15S** **c.139G > A, p.G47S** c.459_490insA	AGS1
F6	P7 [15]^b^	*TREX1*	c.294dupA, p.C99fs c.868_885del, p.P290_A295del	AGS1
F7	P8 [16]^c^	*RNASEH2B*	c.859G > T, p.A287S, Hom **c.269C > T, p.P90L, Het**	AGS2
F8	P9 [17] Brother of P10	*RNASEH2B*	c.294_295insA, p.C99MfsX2 c.868_885del, p.290_295delaa	AGS2
F8	P10 [17] Sister of P9	*RNASEH2B*	c.294_295insA, p.C99MfsX2 c.868_885del, p.290_295delaa	AGS2
F9	P11^c^	*RNASEH2C*	**c.194G > A, p.G65D** **c.427A > G, p.K143E**	AGS3
F10	P12^c^	*RNASEH2C*	c.227C > T, p.P76L c.194G > A, p.G65D	AGS3
F11	P13^c^	*RNASEH2C*	c.461C > T, p.A154V c.197G > A, p.R66H	AGS3
F12	P14 [18]	*RNASEH2A*	c.199G > C, p.D67H c.322C > T, p.R108W	AGS4
F13	P15^a,d^	*ADAR1*	c.305_306del, p.Q102Rfs*22	AGS6
F14	P16 [19]^e^	*ADAR1*	c.1A > G, p.M1V c.3124C > T, p.R1042C	AGS6
F15	P17 [20]^a^	*IFIH1*	**c.1016C > A, p.A339D**	AGS7
F16	P18 [21]^f^	*IFIH1*	c.2336G > A, p.R779H	AGS7
F17	P19 [22]	*IFIH1*	c.2336G > A, p.R779H	AGS7
F18	P20^c^	*IFIH1*	c.2336G > A, p.R779H	AGS7
F19	P21^a^	*IFIH1*	c.2336G > A, p.R779H	AGS7
F20	P22^a,g^ Brother of P23	*IFIH1*	**c.2131C > A, p.Q711K**	AGS7
F20	P23^a,g^ Sister of P22	*IFIH1*	**c.2131C > A, p.Q711K**	AGS7

New mutations are labelled in bold. *TREX1* three prime repair exonuclease 1, *RNASEH2A* ribonuclease H2 subunit A, *RNASEH2B* ribonuclease H2 subunit B, *RNASEH2C* ribonuclease H2 subunit C, *ADAR1* adenosine deaminase acting on RNA, *IFIH1* interferon induced with helicase C domain 1, *AGS* Aicardi-Goutieres syndrome. ^a^These case were from the Department of Pediatrics, Peking Union Medical College Hospital; ^b^the sister of the patient had the same mutation, only showing a chilblain-like rash; ^c^this case was from the Department of Rheumatology and Immunology, Shenzhen Children's Hospital; ^d^the father of the patient had the same mutation, showing only a chilblain-like rash; ^e^the brother of the patient had the same mutation, only showing symmetric pigment abnormalities; ^f^the mother and small brother of the patient had the same mutation but no clinical symptoms; ^g^the mother of the patient had the same mutation but no clinical symptoms

### Molecular data

Six AGS-related genes were involved. Eleven *TREX1* mutations, 4 *RNAHSEH2B* mutations, 5 *RNAHSEH2C* mutations, 2 *RNAHSEH2A* mutations, 3 *ADAR1* mutations, and 3 *IFIH1* mutations were identified in 23 patients. A total of 13 new mutations were identified.

### Clinical features

The clinical manifestations of the 23 cases of AGS are detailed in Table 4, including 11 male and 12 female patients. The median age of onset was 6 months (0–132 months), and 82.6% experienced onset before 3 years of age. Two cases were prenatal onset AGS, 12 were infantile onset AGS and 9 were later onset AGS. No patients with bilateral striatal necrosis, hereditary spastic paraparesis, and *SAMHD1*-related cerebrovascular disease were reported in our study. The mean age at the last follow-up was 8.3 years, ranging from 5 months to 39 years. One of the most commonly affected organ systems in AGS is the central nervous system (100%), which can cause disabling neurological manifestations, including dystonia, epilepsy, and retardation of mental, motor and language functions. Images of the head showed intracranial calcification, leukodystrophy and brain atrophy. A total of eight patients showed epileptic symptoms, seven of whom were treated with antiepileptic drugs, four of whom had no further seizures after treatment, and three were poorly controlled. A small number of patients presented with cerebrovascular abnormalities. In addition, five children had feeding difficulties due to severe neurological symptoms, three of which had long-term gastric tubes. Skin lesions occurred in 60.87% of patients, and chilblain-like rash was the main manifestation. Other types of rash, such as livedo, erythaema nodule, and Raynaud's phenomenon, were found in a few cases. The disease also led to physical retardation, such as microcephaly (47.62%) and short stature (52.38%). Other affected organs included the liver, thyroid, blood system, lungs, kidneys and joints. Thyroid involvement manifested as subclinical hypothyroidism and clinical hypothyroidism; lung involvement presented as interstitial lung disease.

**Table 4:** Manifestations of cases with Aicardi-Goutieres syndrome

Clinical symptoms	Percentage, %
Fever	30.43 (7/23)
Skin	60.87 (14/23)
Chilblain-like rash	47.83 (11/23)
Livedo	4.35 (1/23)
Erythaema nodosa	4.35 (1/23)
Raynaud's phenomenon	4.35 (1/23)
Telangiectasia	4.35 (1/23)
Nervous system	100 (23/23)
Intracranial calcification	100 (21/21)
Dystonia	60.87 (14/23)
Leukodystrophy	54.17 (13/21)
Epilepsy	34.78 (8/23)
Brain atrophy	38.10 (8/21)
Microcephaly	47.62 (10/21)
Short stature	52.38 (11/21)
Mental retardation	57.14 (12/21)
Motor skill impairment	60.87 (14/23)
Language disability	42.86 (9/21)
Interstitial lung disease	19.05 (4/21)
Urinary system	15.00 (3/20)
Chronic renal interstitial lesions	10.00 (2/20)
Membrane hyperplasia and membranous nephropathy	5.00 (1/20)
Arthralgia	13.04 (3/23)
Haematological system	21.05 (4/19)
Anaemia	5.26 (1/19)
Neutropaenia	5.26 (1/19)
Thrombocytopaenia	5.26 (1/19)
Lymphopaenia	5.26 (1/19)
Hypohepatia	42.11 (8/19)
Splenomegaly	9.52 (2/21)
Thyroid dysfunction	46.15 (6/13)
Subclinical hypothyroidism	30.77 (4/13)
Hypothyroidism	15.38 (2/13)
Elevated ESR	53.85 (7/13)
Elevated CRP	15.38 (2/13)
Decreased C3 complement	36.36 (4/11)
Decreased C4 complement	36.36 (4/11)
Positive autoimmune antibodies	66.67 (8/12)
Anti-nuclear antibody	50.00 (6/12)
Anti-double-stranded DNA antibody	27.27 (3/11)
Rheumatoid factor	27.27 (3/11)
Anti-neutrophil cytoplasmic antibody	20.00 (2/10)
Anti-Sjogren syndrome A antibody	9.09 (1/11)
Anti-Sjogren syndrome B antibody	9.09 (1/11)
Mortality	4.35 (1/23)

*ESR* erythrocyte sedimentation rate, *CRP* C-reactive protein

Fever occurred in 30.43%. The fever-type was irregular and the fever caused by the infection was excluded. Two patients with fever were treated with glucocorticoid and their fever were not controlled. Elevated erythrocyte sedimentation rate in 53.85%, hypocomplementaemia in 36.36% and autoimmune antibody positivity in 66.67%, included antinuclear antibody (ANA), anti-double-stranded DNA (ds-DNA) antibody, rheumatoid factor, and anti-neutrophil cytoplasmic antibody, among others. P2 (AGS1), for whom onset was at the age of 3 years that manifested as a chilblain-like rash, repeated cerebral infarction, and ANA and anti-ds-DNA antibody positivity, was diagnosed with systemic lupus erythaematosus (SLE) and with AGS1 based on gene sequencing. P15 (AGS6), who had onset at 3 years of age and presented with a chilblain-like rash, cytopaenia, epilepsy, ANA and ds-DNA antibody positivity and hypocomplementaemia, was also diagnosed with SLE. However, traditional treatment, such as glucocorticoids, hydroxychloroquine, cyclophosphamide, and mycophenolate mofetil, were ineffective. P15 was later diagnosed with AGS6 by gene sequencing.

Three families involved 2 patients, including F4 (P4 and P5), F8 (P9 and P10), and F20 (P22 and P23). The age of onset, severity, and disease progression varied between individuals within the same families, even if patients shared the same mutation. In addition, the sister of P7, the father of P15, the brother of P16, the mother and brother of P20 and the mother of P22 harboured the same mutation as the patients, with only skin lesions or no clinical symptoms (Table 2).

Some neurological sequelae were present in our patients. A total of 12 cases manifested as cognitive impairment of developmental delay, 14 as motor skill impairment and 9 as language disability. One (4.35%) patient died due to uraemia caused by severe renal interstitial lesions.

### Interferon-stimulated genes expressions

**Fig. 1:**
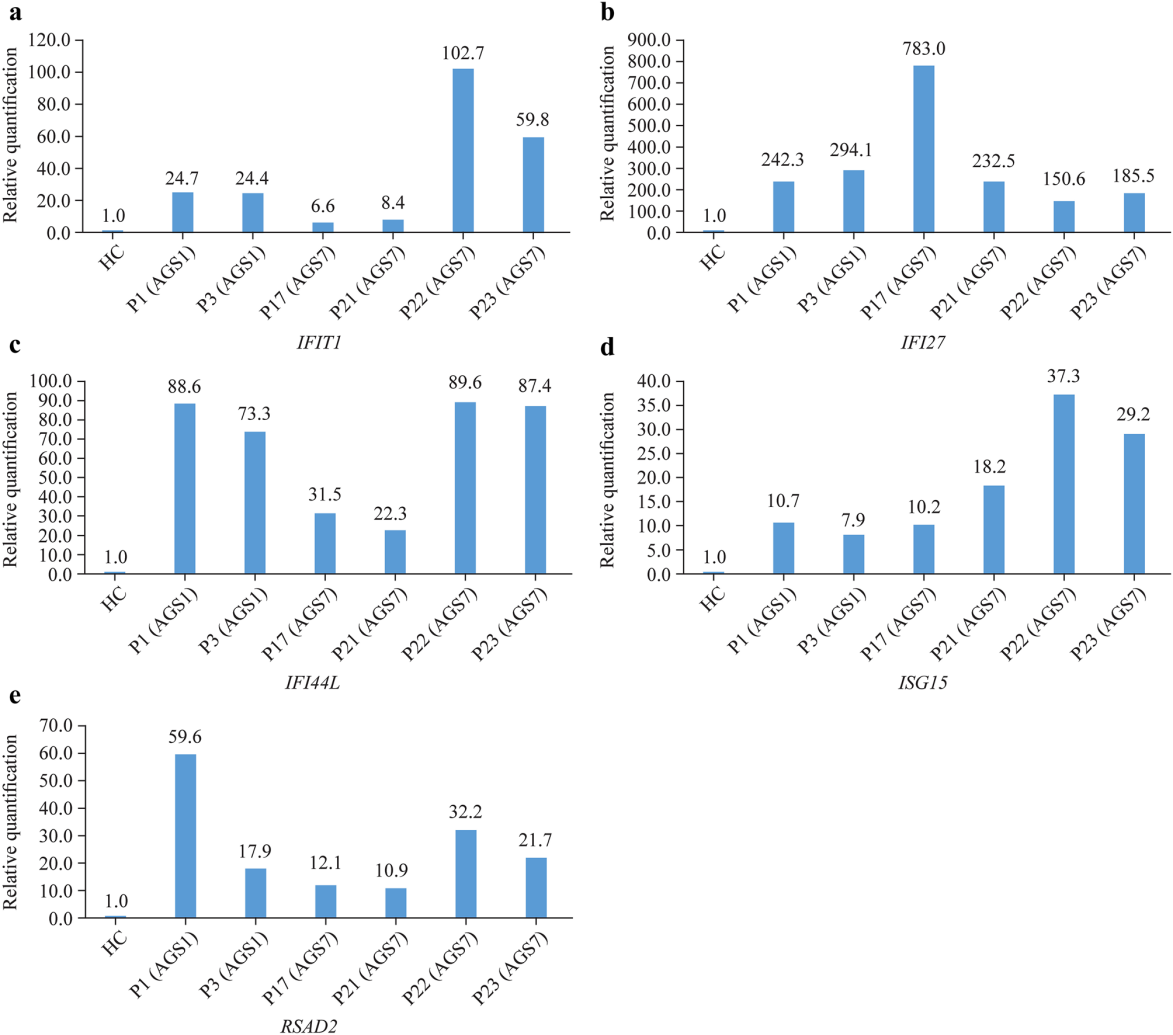
Real-time polymerase chain reaction detection of relative expression of *ISGs* in six patients, including two with AGS1 (P1 and P3) and four with AGS7 (P17, P21, P22 and P23). **a** *IFIT1*; **b** *IFI27*; **c** *IFI44L*; **d** *ISG15*; **e** *RSAD2*. *IFI* interferon alpha-inducible protein encoding gene, *ISGs* interferon-stimulated genes*, RSAD2* radical S-adenosyl methionine domain containing 2 encoding gene

Real-time PCR was used to detect relative expression of *ISGs* in six patients, including two with AGS1 (P1 and P3) and four with AGS7 (P17, P21, P22 and P23). RQ of *ISGs* were elevated, which was dominated by *IFI27* (Fig. 1).

## Discussion

AGS is a childhood autoinflammatory disease characterized by constitutive upregulation of type I interferon, referred to as type I interferonopathies [23]. Activation of the type I interferon signalling pathway results in increased expression of many *ISGs* [24]. By summarizing and analysing the 23 cases of AGS in China, we herein present the clinical characteristics of Chinese patients and provide clues for clinical identification of AGS. Moreover, 13 novel mutations were found, further enriching the gene mutation spectrum of the disease and providing a reliable basis for genetic diagnosis. Previous studies on AGS in China are mainly case reports, and this is the first study to systematically analyse the characteristics of Chinese patients with AGS. However, the sample size was not large enough to perform effective statistical analysis and find the relationship between genotype and phenotype. Ten cases in our study obtained from a literature review involved incomplete clinical data, which may have influenced the results of the analysis.

To date seven pathogenic genes of AGS have been reported. According to a large-sample study abroad, the most common type is AGS2, accounting for 36%, followed by AGS1, accounting for 22% [2]. While the most common types were AGS1 and AGS7 in this study. The difference may be associated with ethnicity or small sample size.

AGS was defined as a neurological disease with an early age of onset and a progressive course [1]. Over the years, some features of the disease have been clarified: the disease is characterized by a first subacute phase that children affected show the neurological deterioration, followed by a more chronic course [25]. Typical AGS is characterized by severe neurological symptoms, such as dystonia, seizures, developmental delay or motor regression, pyramidal signs, cognitive impairment of developmental delay, language or speech disorder, intracranial calcification, leukodystrophy, and brain atrophy [3, 26, 27]. Chilblain-like rash is another important clinical feature of AGS [2]. With the increase in the number of cases, we recognize that AGS is a disease involving multiple organs [2, 6], causing liver damage, cytopaenia, lung interstitial lesions, and abnormalities in thyroid and kidney functions. In this study, the clinical manifestations of multiple organs of patients with AGS in China were basically the same as those previously reported in other countries.

AGS is an autoinflammatory disease sharing many features with autoimmune diseases such as SLE. In 2010, a study abroad that included 26 cases of AGS reported that 46.15% of cases exhibited features of SLE, such as thrombocytopaenia, leukopaenia, antinuclear antibodies, and erythaematous lesions [28]. In our study, autoimmune antibody positivity was detected in more than 60% of patients with AGS, which is significantly higher than in reports from other countries. The proportion of immune system abnormalities in our study was higher than previously reported, which is considered to be related to selection bias. More than half of the patients we enrolled were from the outpatient clinic of rheumatology, whereas patients in previous studies were mostly from the outpatient clinic of the neurology department. In addition, two cases in our study met the diagnostic criteria of SLE, both involving onset at the age of 3 and manifesting as chilblain-like rash and intracranial calcification. One of these patients was treated with glucocorticoids and multiple immunosuppressants, which were ineffective. The peak incidence of childhood-onset SLE occurs around puberty and predominantly involves females [29]. Skin and mucosal involvement can appear as butterfly-shaped facial and cutaneous rashes, disc erythema, and oral ulcers [30], and neurological manifestations include headache, neuropsychiatric symptoms, cerebrovascular disease, and epilepsy [31]. Glucocorticoid and immunosuppressive agents are classic treatments of SLE. Therefore, rheumatic disease with early onset may be a diagnostic clue for AGS, especially for patients with atypical clinical manifestations who do not respond to classic treatments.

Incomplete penetrance and marked intrafamilial clinical variability in AGS are observed. In our study, family members with identical mutations had diverse clinical manifestations and different disease severities, which has already been reported [6] and may be related to environmental factors and epigenetics. Therefore, the diagnosis of AGS depends on both clinical symptoms and genetic test results.

In conclusion, AGS has a broad clinical disease spectrum with heterogeneity and incomplete penetrance, and its diagnosis depends on both clinical manifestations and genetic results. Overall, the central nervous system involvement of AGS affects quality of life, and there is a high rate of mortality. Early identification and early treatment have important clinical significance. For patients with early onset of chilblain-like rash, neurological symptoms (intracranial calcification, white matter lesions, abnormal muscle tone), delayed development, positive autoimmune antibodies, and atypical clinical manifestations of SLE or who do not respond to conventional treatments of SLE, AGS should be considered. ISGs and genetic testing can be used to confirm diagnosis. It is also necessary to actively evaluate the function of the thyroid, blood, liver, kidney, lung and other organs and provide timely symptomatic treatment.

## Data Availability

The datasets generated and/or analyzed during the current study are available from the corresponding author on reasonable request.
